# Association of Socioeconomic Changes due to the COVID-19 Pandemic With Health Outcomes in Patients With Skin Diseases: Cross-Sectional Survey Study

**DOI:** 10.2196/22288

**Published:** 2020-09-11

**Authors:** Yeye Guo, Minxue Shen, Xu Zhang, Yi Xiao, Shuang Zhao, Mingzhu Yin, Wenbo Bu, Yan Wang, Xiang Chen, Juan Su

**Affiliations:** 1 Department of Dermatology Xiangya Hospital Central South University Changsha China; 2 National Clinical Research Center for Geriatric Disorders Xiangya Hospital Changsha China; 3 Hunan Engineering Research Center of Skin Health and Disease Hunan Key Laboratory of Skin Cancer and Psoriasis, Xiangya Hospital Central South University Changsha China; 4 Department of Social Medicine and Health Management Xiangya School of Public Health Central South University Changsha China; 5 Institute of Dermatology Chinese Academy of Medical Science and Peking Union Medical College Nanjing China

**Keywords:** skin diseases, coronavirus disease 2019, unemployment, quality of life, web-based, survey, dermatology, COVID-19, lifestyle, impact, outcome, isolation

## Abstract

**Background:**

The outbreak of COVID-19 has profoundly influenced people’s lifestyles; these impacts have varied across subgroups of people. The pandemic-related impacts on the health outcomes of people with dermatological conditions are unknown.

**Objective:**

The aim of this paper was to study the association of COVID-19 pandemic–related impacts with health-related quality of life in patients with skin diseases.

**Methods:**

This was a cross-sectional study among Chinese patients with skin diseases. A self-administered web-based questionnaire was distributed through social media. Demographic and clinical data and pandemic-related impacts (isolation status, income changes, and employment status) were collected. The main outcomes included perceived stress (Visual Analog Scale), symptoms of anxiety (Generalized Anxiety Disorder-7) and depression (9-Item Patient Health Questionnaire), quality of life (Dermatology Life Quality Index), and health utility mapping based on the EQ-5D-3L descriptive system. Multivariable logistic regression was used to investigate the associations.

**Results:**

A total of 506 patients with skin diseases completed the survey. The mean age of the patients was 33.5 years (SD 14.0), and 217/506 patients (42.9%) were male. Among the 506 respondents, 128 (25.3%) were quarantined, 102 (20.2%) reported unemployment, and 317 (62.6%) reported decrease or loss of income since the pandemic. The pandemic-related impacts were significantly associated with impaired mental well-being and quality of life with different effects. Unemployment and complete loss of income were associated with the highest risks of adverse outcomes, with increases of 110% to 162% in the prevalence of anxiety, depression, and impaired quality of life.

**Conclusions:**

Isolation, income loss, and unemployment are associated with impaired health-related quality of life in patients with skin diseases during the COVID-19 pandemic.

## Introduction

The outbreak of COVID-19 has resulted in the infection of over 4,000,000 people in 216 countries and regions worldwide as of May 17, 2020 [1]. A global response of social distancing was undertaken to control the transmission of the disease, and people started voluntarily isolating themselves at home. The Chinese government suggested that the public quarantine at home in January 2020. In the following months, many other countries, such as the United Kingdom, Italy, and the United States, called for self-isolation [2,3]. These actions effectively reduced the rapid spread of the disease but unavoidably had a substantial impact on the global economy. During this time, great socioeconomic changes occurred, such as reduced workforce and increasing unemployment [4]. According to data from the US Labor Department, the unemployment rate soared to 14.7% in April 2020 [5]. According to a survey in the United States, up to 43.4% of adults reported that their families suffered a job or income loss during the pandemic [6].

In addition to the socioeconomic implications of the pandemic, the health status of the public is a critical issue that should not be ignored. It is well-known that isolation itself is a risk factor for many mental health issues, including suicide and self-harm [2]. Therefore, long-term isolation in conjunction with loss of employment or income may produce adverse effects on individuals’ mental well-being and quality of life [7], especially for people who have chronic diseases.

Skin disorders are among the most prevalent human diseases; they affect 30% to 70% of individuals [8]. Skin diseases may cause disfigurement and cutaneous symptoms, and they may result in considerable discomfort and disability. Although most skin diseases are not life-threatening, many of these diseases, such as psoriasis and eczema, are chronic and recurrent. Additionally, itch and fatigue are common symptoms reported by patients with skin diseases, and some also report experiencing pain [9]. Consequently, skin diseases cause not only physical but also psychological discomfort as well as impaired quality of life [10]. Additionally, skin diseases create financial burdens on families and society. A study in the United States suggested that skin diseases cost an estimated $39.3 billion per year [11]. As a result, isolation, unemployment, and loss of income related to the COVID-19 pandemic may place patients with skin diseases at additional health risk.

To date, no study has been published regarding the potential influence of socioeconomic changes on the health status of patients with skin diseases during the COVID-19 pandemic. In this study, we investigated the association of COVID-19–related impacts, including isolation, unemployment, and income loss, with health-related quality of life in patients with skin diseases, including perceived stress, symptoms of anxiety and depression, and quality of life during the pandemic.

## Methods

### Study Design and Participants

We performed a cross-sectional study among Chinese patients with skin diseases. A web-based survey link was created and posted on social media platforms (WeChat groups and teledermatology platforms) to facilitate the collection of questionnaires. To avoid repeat submissions, each single IP address was allowed to submit answers only one time. Completion of all questions was required before submission of the questionnaire. All the participants were allowed to quit at any time if any question made them feel uncomfortable. The survey was conducted from April 15 to 27, 2020. The study was reviewed and approved by the institutional research ethics board of Xiangya Hospital, Central South University (Changsha, China; approval number: 202002024). Electronic informed consent was collected from all participants.

### Exposure Variables

Three exposure variables were defined. Outdoor activity restriction was determined by a single question: “During the last month, what measures did you take for isolation?” with the following four responses: “I was not isolated, and my outdoor activity was unaffected,” “I was not isolated, but my outdoor activity was partly affected,” “I was isolated at home and receiving medical observation,” and “I was quarantined in hospital and receiving medical observation or treatment.” Because only one patient reported having COVID-19 and being quarantined in hospital, patients who were quarantined at home or in the hospital were combined into one group in the analysis.

The employment status was determined by a single question: “How is your employment status since the epidemic?” with the following responses: “I am unemployed since the epidemic” and “My employment status has been unaffected since the epidemic.”

Income change was measured by a single question: “Has your monthly income changed since the epidemic of COVID-19?” with the following responses: “I have completely lost my income,” “My monthly income has decreased,” “My monthly income has been unaffected,” and “My monthly income has increased.” Because only two patients reported increased income, they were categorized in the “unaffected” group in the analysis.

### Patient-Reported Outcomes

The primary outcomes were perceived stress and depression and anxiety, which were measured using some short, simple scales to minimize respondent burden.

The Visual Analog Scale (VAS) was used to assess perceived stress during the past two weeks. The area under the receiver operating characteristic curve of the VAS was 0.9 to 0.93, with a cutoff of 6.8 to 7.2 according to the Perceived Stress Scale-14 (PSS-14) [12,13]. Here, we used a cutoff of ≥7 to define significant perceived stress.

The Generalized Anxiety Disorder-7 (GAD-7) and the 9-item Patient Health Questionnaire (PHQ-9) were applied to examine the symptoms of anxiety and depression during the past two weeks. The cutoff point for both scales was ≥8 [14,15]. The Cronbach α coefficients of the GAD-7 and PHQ-9 in our sample were .93 and .89, respectively.

The Dermatology Life Quality Index (DLQI) was used to assess the respondents’ quality of life during the past two weeks. The cutoff point was ≥10 in our study [16]. The Cronbach α coefficient of the DLQI in our sample was .76. Health utility was mapped using the generic tool EQ-5D-3L according to the method proposed by Liu et al [17].

### Covariates

Demographic and clinical data of the individuals were collected and analyzed as covariates, including gender (male or female), age, marital status (unmarried, married, divorced, or widowed), educational level (primary school and below, middle school, high school, or college and above), annual income (<¥10,000, ¥10,000 to ¥49,999, ¥50,000 to ¥99,999, or ≥¥100,000, equivalent to US $1,464.47, $1,464.4 to $7,322.21, $7,322.36 to $14,644.57, or ≥$14,644.72), type of skin diseases (infectious skin diseases, papulosquamous disorders, allergic skin diseases, disorders of appendages, pigmentary disorders, skin tumors), course of disease (<1 year, 1 to 5 years, 6 to 10 years, >10 years), adherence to treatment, and use of health care services.

### Statistical Analyses

The data were exported from the web-based survey system and analyzed with R version 3.4 (R Project). Continuous variables with normal distribution were expressed as mean (SD) and compared with analysis of variance. Continuous data with skewed distributions were presented as median (IQR) and compared with the Wilcoxon rank sum test. Categorical variables were summarized as counts (percentages) and compared using the chi-square test or Fisher exact test. The interaction effects of the exposure variables were examined. The effect sizes of the associations were presented as adjusted odds ratios (aORs) and 95% CIs. A *P* value <.05 was considered statistically significant. Reporting of the results followed the Strengthening the Reporting of Observational studies in Epidemiology (STROBE) guidelines.

### Data Accessibility

Data are available upon request from JS.

## Results

In total, 506 valid questionnaires were collected and analyzed. No patient reported confirmed infection with COVID-19. The average time to complete the survey was 7.2 minutes (IQR 4.1-8.2). The mean age of the patients was 33.5 years (SD 14.0), and 217/506 patients (42.9%) were men. As shown in Table 1, allergic skin diseases are the most commonly reported type (103/506, 20.4%), followed by papulosquamous disorders (58/506, 11.5%), infectious skin diseases (57/506, 11.3%), disorders of appendages (46/506, 9.1%), pigmentary disorders (38/506, 7.5%), hair disorders (38/506, 7.1%), and skin tumors (30/506, 5.9%).

Of the 506 participants, 252 (49.8%) reported unaffected outdoor activity, 126 (24.9%) reported partly restricted outdoor activity, and 128 (25.3%) reported isolation. As shown in Table 2, outdoor activity restriction was significantly associated with anxiety, depression, and impaired quality of life (all *P*<.05). A total of 102/506 patients (20.2%) reported unemployment, and approximately two-thirds of the participants (317/506, 62.6%) experienced decrease or loss of income during the pandemic. Both decreased income and unemployment were significantly associated with perceived stress, symptoms of anxiety and depression, and impaired quality of life (all *P*<.05).

**Table 1:** Characteristics of the participants (N=506).

Characteristic		Value
Age (years), mean (SD)		33.5 (14.0)
**Gender, n (%)**		
	Male	217 (42.9)
	Female	289 (57.1)
**Marital status, n (%)**		
	Unmarried	189 (37.4)
	Married	291 (57.5)
	Widowed	22 (4.3)
	Divorced	4 (0.8)
**Educational level, n (%)**		
	Primary school and below	49 (9.7)
	Middle school	69 (13.6)
	High school	82 (16.2)
	College and above	306 (60.5)
**Income (¥)** ^a^ **, n (%)**		
	<10,000	155 (30.6)
	10,000-49,999	132 (26.1)
	50,000-99,999	122 (24.1)
	≥100,000	97 (19.2)
**Skin disease, n (%)**		
	Infectious skin diseases	57 (11.3)
	Papulosquamous disorders	58 (11.5)
	Allergic skin diseases	103 (20.4)
	Disorders of appendages	46 (9.1)
	Disorders of hairs	36 (7.1)
	Pigmentary disorders	38 (7.5)
	Skin tumors	30 (5.9)
	Other	189 (37.4)
**Course of disease (years), n (%)**		
	<1	202 (39.9)
	1-5	165 (32.6)
	6-10	57 (11.3)
	>10	82 (16.2)
**Isolation, n (%)**		
	Outdoor activity unrestricted	252 (49.8)
	Outdoor activity partly restricted	126 (24.9)
	Isolated at home or in hospital	128 (25.3)
**Loss of income, n (%)**		
	Unaffected	189 (37.4)
	Reduced	208 (41.1)
	Completely lost	109 (21.5)
**Adherence to treatment, n (%)**		
	Adherent to treatment	121 (23.9)
	No treatment prescribed	210 (41.5)
	Not adherent to treatment	175 (34.6)
**Use of health care services, n (%)**		
	Visited a physician in hospital	126 (24.9)
	Consulted a doctor remotely	92 (18.2)

^a^1 ¥ = US $0.15.

**Table 2:** Associations of epidemic-related impacts with patient-reported outcome scores of skin diseases (N=506).

Exposure group		Stress VAS^a^			PHQ-9^b^			GAD-7^c^			DLQI^d^			Health utility		
		Mean SE		*P* value	Mean SE		*P* value	Mean SE		*P* value	Mean SE		*P* value	Mean SE		*P* value
**Isolation**																
	Unaffected	4.05 (0.18)		Ref ^e^	4.56 (0.30)		Ref	3.31 (0.26)		Ref	5.47 (0.41)		Ref	0.96 (0.01)		Ref
	Restricted	4.56 (0.26)		.10	6.33 (0.42)		<.001	4.97 (0.37)		.001	6.13 (0.58)		.35	0.93 (0.01)		.02
	Isolated	4.38 (0.25)		.30	5.77 (0.42)		.02	4.20 (0.37)		.049	7.11 (0.58)		.02	0.93 (0.01)		.02
**Loss of income**																
	Unaffected	3.23 (0.20)		Ref	4.17 (0.34)		Ref	3.19 (0.30)		Ref	4.85 (0.47)		Ref	0.96 (0.01)		Ref
	Reduced	4.54 (0.19)		<.001	5.77 (0.32)		.001	4.19 (0.29)		.02	6.18 (0.45)		.04	0.94 (0.01)		.051
	Completely lost	5.50 (0.26)		<.001	6.39 (0.45)		<.001	4.79 (0.40)		.002	7.87 (0.62)		<.001	0.92 (0.01)		<.001
**Unemployment**																
	Unaffected	3.91 (0.14)		Ref	4.84 (0.23)		Ref	3.60 (0.21)		Ref	5.28 (0.32)		Ref	0.95 (0.00)		Ref
	Unemployed	5.64 (0.28)		<.001	7.15 (0.46)		<.001	5.30 (0.41)		<.001	9.10 (0.63)		<.001	0.91 (0.01)		<.001
**Adherence to treatment**																
	Adherent	4.75 (0.26)		Ref	5.64 (0.43)		Ref	3.59 (0.38)		Ref	6.24 (0.58)		Ref	0.93 (0.01)		Ref
	No treatment needed	3.94 (0.20)		.01	4.41 (0.32)		.02	3.30 (0.29)		.54	4.12 (0.44)		<.001	0.97 (0.01)		<.001
	Nonadherent	4.30 (0.22)		.18	6.14 (0.36)		.37	4.97 (0.31)		.005	8.23 (0.48)		.008	0.93 (0.01)		.75

^a^VAS: Visual Analog Scale.

^b^PHQ-9: Patient Health Questionnaire-9.

^c^GAD-7: Generalized Anxiety Disorder-7.

^d^DLQI: Dermatology Life Quality Index.

^e^ Ref: reference group.

We further categorized the scale scores by clinically relevant cutoffs and performed a series of logistic regression models with adjustments. As shown in Table 3, outdoor activity restriction was significantly associated with increased symptoms of depression (aOR 1.36-1.81) and anxiety (aOR 1.39-2.20) as well as impaired quality of life (aOR 1.22-1.78) in a dose-dependent manner (quarantined > partly restricted compared with unrestricted); however, it was not significantly associated with stress. Loss of income was correlated with stress (aOR 1.59-4.05), depression (aOR 2.56-2.56), anxiety (aOR 1.64-2.48), and impaired quality of life (aOR 1.27-2.62) in a dose-dependent manner (loss of income > reduced income compared with unaffected income). Similarly, unemployment was significantly associated with adverse outcomes, including perceived stress, depression, anxiety, and impaired quality of life.

**Table 3:** Associations of epidemic-related impacts with patient-reported outcomes of skin diseases (N=506).

Exposure group		Perceived stress (VAS^a^ ≥7)			Depression (PHQ-9^b^ ≥8)			Anxiety (GAD-7^c^ ≥8)			Impaired quality of life (DLQI^d^ ≥10)		
		aOR^e,f^	95% CI	*P* value	aOR	95% CI	*P* value	aOR	95% CI	*P* value	aOR	95% CI	*P* value
**Isolation**													
	Unaffected	Ref^g^	N/A^h^	N/A	Ref	N/A	N/A	Ref	N/A	N/A	Ref	N/A	N/A
	Restricted	0.90	0.52-1.56	.71	1.36	0.84-2.21	.21	1.39	0.73-2.64	.31	1.22	0.70-2.12	.48
	Isolated	1.02	0.59-1.77	.93	1.81	1.13-2.89	.013	2.20	1.23-3.96	.008	1.78	1.05-3.01	.03
**Loss of income**													
	Unaffected	Ref	N/A	N/A	Ref	N/A	N/A	Ref	N/A	N/A	Ref	N/A	N/A
	Reduced	1.59	0.91-2.79	.10	2.22	1.38-3.57	.001	1.64	0.88-3.06	.12	1.27	0.73-2.20	.40
	Complete loss	4.05	2.13-7.72	<.001	2.56	1.43-4.58	.002	2.48	1.19-5.13	.02	2.62	1.40-4.92	.003
**Unemployment**													
	Unaffected	Ref	N/A	N/A	Ref	N/A	N/A	Ref	N/A	N/A	Ref	N/A	N/A
	Unemployed	2.41	1.42-4.08	.001	2.11	1.30-3.43	.003	2.60	1.45-4.65	.001	2.59	1.54-4.35	<.001
**Adherence to treatment**													
	Adherent	Ref	N/A	N/A	Ref	N/A	N/A	Ref	N/A	N/A	Ref	N/A	N/A
	No treatment needed	0.81	0.461.41	.45	0.86	0.52-1.44	.26	0.66	0.33-1.29	.22	0.58	0.32-1.04	.07
	Nonadherent	0.81	0.46-1.42	.46	1.39	0.84-2.31	.68	1.39	0.75-2.58	0.297	1.35	0.78-2.33	.29

^a^VAS: Visual Analog Scale.

^b^PHQ-9: Patient Health Questionnaire-9.

^c^GAD-7: Generalized Anxiety Disorder-7.

^d^DLQI: Dermatology Life Quality Index.

^e^aOR: adjusted odds ratio.

^f^Adjusted for age, gender, income, and educational level.

^g^Ref: reference group.

^h^N/A: not applicable.

Among the 506 participants, 175 (34.6%) reported nonadherence to treatment. The reasons for nonadherence included limited accessibility to health care due to isolation (n=273, 53.9%), voluntarily stopping a prescription (n=106, 20.9%), forgetting to take medicine (n=71, 14.0%), and limited accessibility to medications (n=56, 11.1%). However, nonadherence was not significantly associated with adverse outcomes (Tables 2 and 3).

## Discussion

### Principal Findings

In the current study, we investigated the association of the impact of the COVID-19 pandemic with health-related quality of life in Chinese patients with skin diseases through a web-based survey. Our results indicated that over half of the respondents experienced quarantine and loss of income during the pandemic. These patients reported higher levels of perceived stress, increased symptoms of anxiety and depression, and impaired quality of life. To the best of our knowledge, this is the first impact analysis of the COVID-19 pandemic in patients with skin diseases. Our study reveals considerable proportions of impaired quality of life and mental well-being in these patients. Telemedicine, mental health intervention, and social support are needed for patients with skin diseases during this particular period.

Studies have indicated increases in mental health issues among medical staff [18] and patients with chronic diseases [19] since the outbreak of COVID-19. However, there is little research regarding the impact of the pandemic on patients with skin diseases. Here, we identified isolation, unemployment, and loss of income as significant risk factors for poor mental well-being and quality of life in patients with skin diseases.

Public health efforts to prevent the spread of COVID-19 are having a growing impact on the global economy. A considerable number of individuals are losing their jobs, at least temporarily [20]. It has been estimated that the worldwide unemployment rate will increase from 4.936% to 5.644% due to the COVID-19 pandemic [2]. According to our data, over 20% of participants (109/506, 21.5%) became unemployed, which is notably higher than the reported unemployment rate. Additionally, 208 of the 506 patients (41.1%) experienced reduced income even though they were not unemployed; this also resulted in impaired mental health and quality of life. In addition to the financial impacts, job loss can disrupt health insurance coverage [21]. Although many countries have provided free testing or even treatment for COVID-19, medical costs for other conditions related to the pandemic are generally not covered by these reimbursement policies.

It should be noted that 65% of the respondents’ nonadherence to treatment was related to limited accessibility to health institutions or medications based on our observations. Since the pandemic, many hospitals and health care institutions have temporarily closed their outpatient services to avoid nosocomial infection and better allocate health care resources, which has created unique challenges to health care delivery at present [22]. A previous study described the possibility of using telemedicine in disasters or public health emergencies [23]. Most developed areas have been using telemedicine not only for physical health care, but also for mental health services [24]. However, it is difficult for patients in rural areas who lack internet services and smartphones to use remote health care. Current telemedicine programs in China and some countries enable consultation services but do not provide medication delivery services; this could eventually cause nonadherence to treatment due to lack of medication.

### Limitations

Our study has some limitations. First, the survey was web-based, with low representativeness due to nonprobability sampling. Second, the exposure and outcome variables were self-reported, and recall bias may have been introduced. Third, some relevant outcomes such as sleep quality were not included in our study because a heavy survey burden may lead to fewer responses and lower accuracy. Last, the survey was conducted among Chinese patients and may not fully represent all patients in areas beyond China due to cultural differences as well as variations in reimbursement policies and social systems.

### Conclusions

Taken together, our findings indicate that pandemic-related impacts are associated with adverse patient-reported outcomes of skin diseases. Early and timely mental health intervention, telemedicine, and health education are needed for these patients. Preferably, social support and reimbursement policies will further help patients under heavy financial burdens endure this difficult period.

## Abbreviations

aOR: adjusted odds ratio
DLQI: Dermatology Life Quality Index
GAD-7: Generalized Anxiety Disorder-7
PHQ-9: 9-Item Patient Health Questionnaire
PSS-14: Perceived Stress Scale-14
STROBE: Strengthening the Reporting of Observational studies in Epidemiology
VAS: Visual Analog Scale
